# Age does not influence the disease course in a mouse model of *Streptococcus pneumoniae* serotype 3 meningitis

**DOI:** 10.1186/s12979-018-0129-4

**Published:** 2018-09-07

**Authors:** Anja Manig, Sandra Ribes, Catharina Diesselberg, Stephanie Bunkowski, Roland Nau, Sandra Schütze

**Affiliations:** 1 Institute of Neuropathology, University Medical Center Göttingen, Robert-Koch-Str. 40, 37075 Göttingen, Germany; 2 Department of Clinical Neurophysiology, University Medical Center Göttingen, Robert-Koch-Str. 40, 37075 Göttingen, Germany; 3 Department of Geriatrics, Evangelisches Krankenhaus Göttingen-Weende, An der Lutter 24, 37075 Göttingen, Germany; 4 Department of Geriatrics, AGAPLESION Frankfurter Diakonie Kliniken, Wilhelm-Epstein-Str. 4, 60431 Frankfurt, Germany

**Keywords:** Immunosenescence, Age, Mouse model, Streptococcus pneumoniae, Bacterial meningitis, Survival, CNS

## Abstract

In order to elucidate the causes for the increased mortality of aged patients with bacterial central nervous system (CNS) infections, we compared the course of *Streptococcus pneumoniae* (*S. pneumoniae*) meningitis in aged and young mice. Aged (21.2 ± 3.1 months, *n* = 40) and young (3.2 ± 0.9 months, *n* = 42) C57BL/6N and B6/SJL mice were infected by intracerebral injection of 50–70 CFU *S. pneumoniae* serotype 3 and monitored for 15 days. Aged and young mice did not differ concerning mortality (35% versus 38%), weight loss, development of clinical symptoms, bacterial concentrations in cerebellum and spleen as well as the number of leukocytes infiltrating the CNS. In contrast to results from our geriatric mouse model of *Escherichia coli* (*E. coli*) meningitis, where aged mice showed a higher mortality and an impaired elimination of bacteria, we did not find any differences between aged and young mice after intracerebral infection with *S. pneumoniae* serotype 3. This indicates that the increased susceptibility of aged mice to bacterial CNS infections is pathogen-specific: It appears less prominent in infections caused by hardly phagocytable pathogens with thick capsules like *S. pneumoniae* serotype 3, where the age-related decline of the phagocytic capacity of microglia and macrophages has a minor influence on the disease course.

## Background

As a consequence of increasing life expectancy, the population is ageing. It is estimated that in 2050 in all major areas of the world except Africa nearly one quarter or more of the population will be ≥60 years. Globally, the number of persons aged ≥80 years is projected to more than triple by 2050 and to increase more than seven-fold by 2100 [1]. Infectious diseases play an important role in the increasing group of elderly people and are accompanied by a higher mortality and a more severe course of disease [2, 3]. Bacterial meningitis is an infectious emergency. Elderly patients more often develop severe neurological symptoms like coma, epileptic seizures or focal neurological deficits and show an increased mortality in comparison to younger patients [4, 5]. In persons aged ≥60 years, the incidence of *S. pneumoniae* meningitis is approximately 4 times higher and the relative frequency of *Listeria monocytogenes* meningitis is even 15 times higher compared to persons from 2 to 29 years of age [6]. There is an increasing need to elucidate the causes for the increased incidence and mortality of bacterial CNS infections in the expanding population of the elderly and to identify strategies that can protect the elderly against bacterial CNS infections. In our previously published geriatric mouse model of *E. coli* meningitis, aged mice showed an impaired elimination of bacteria and a reduced systemic cyto−/chemokine response accompanied by a faster development of clinical symptoms, a more pronounced and sustained weight loss and a higher mortality [7]. In vitro, primary murine macrophages and microglial cells showed an age-related decline of their ability to phagocytose *E. coli* [7]. These results suggested, that strategies to increase the phagocytic potential of aged macrophages and microglial cells appear promising for the prevention and therapy of CNS infections in the elderly. Here, we compared the disease course in aged and young mice after intracerebral infection with the most frequent pathogen *S. pneumoniae*.

## Methods

*S. pneumoniae* serotype 3, originally isolated from an adult patient with meningitis (gift from Prof. Dr. M. G. Täuber, Division for Infectious Diseases, University of Bern, Switzerland), was grown on blood agar plates, stored at − 80 °C, and diluted in saline. 40 mice at an age of 21.2 ± 3.1 months (aged mice; 19 BL6/SJL and 21 C57BL/6N) and 42 mice at an age of 3.2 ± 0.9 months (young mice; 25 BL6/SJL and 17 C57BL/6 N) were anesthetized with 2 mg ketamine and 0.2 mg xylazine and infected by injection of 10 μl saline containing 50–70 colony-forming units (CFU) of *S. pneumoniae* serotype 3 into the superficial right frontal cortex and subarachnoidal space through the right frontolateral skull. The inoculum dose of 50–70 CFU of *S. pneumoniae* serotype 3 has been identified to cause death in 50% of young C57BL/6N mice (LD_50_) in previous preliminary experiments. During the acute phase of infection (up to 96 h after infection) mice were monitored every 12 h and later on the 7th, 10th and 14th day. Monitoring included weighing and assignment of a clinical score: 0 (no apparent behavioral abnormality), 1 (moderate lethargy), 2 (severe lethargy), 3 (unable to walk), 4 (dead) [8]. When a mouse was no longer able to walk (clinical score 3), it was killed for ethical reasons. 15 days after infection, all surviving mice were killed by cervical dislocation. 30 h after infection, blood was taken from the retrobulbar plexus and bacterial concentrations were determined by quantitative plating on blood agar plates (detection limit 1000 CFU/ml). After death, brain and spleen were removed. One half of the cerebellum and one half of the spleen were homogenized in 500 μl saline, respectively. Bacterial concentrations in cerebellum and spleen were determined by quantitative plating on blood agar plates (detection limit 100 CFU/ml). The left hemisphere of the brain was fixed in 4% formaline, embedded in paraffin, and 2 μm coronal brain sections were used for chloracetate esterase (CAE) staining (Naphthol-AS-D-chloracetate esterase Kit, Sigma-Aldrich, Germany). Stained sections were semi-quantitatively scored for the number of neutrophilic granulocytes in one high-power field (× 40 objective), respectively, in three superficial meningeal regions and the hippocampal fissure for the number of neutrophilic granulocytes using a meningeal inflammation score (no leukocytes [score 0], < 10 leukocytes [score 1], 10–50 leukocytes [score 2], > 50 leukocytes [score 3], for graphical examples see [7]). Statistical analyses and graphical presentation were performed using GraphPad Prism software 5.0 (GraphPad software, San Diego, California, USA). Parametric data were expressed as means ± standard deviations and compared using the Student’s *t*-test. Non-parametric data were expressed as medians and interquartile ranges and compared using the Mann-Whitney *U*-test. For comparison of survival curves the log-rank test was performed. Correlations were analyzed using Spearman’s rank correlation coefficient. *P* values < 0.05 were considered statistically significant.

## Results

**Fig. 1:**
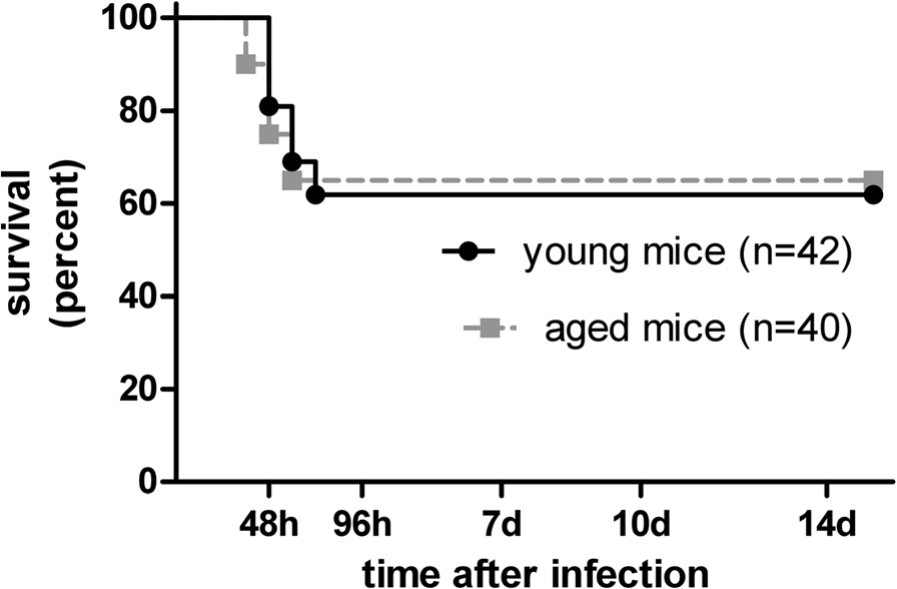
Kaplan-Meier survival curves of young and aged mice after intracerebral infection with 50–70 CFU of *S. pneumoniae* serotype 3

**Fig. 2:**
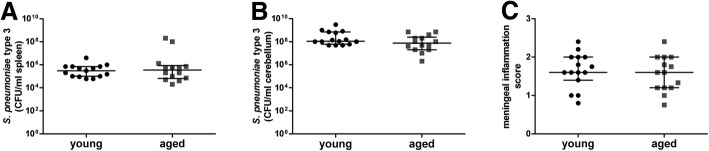
Bacterial concentrations (CFU/ml) in spleen (**a**) and cerebellum (**b**) as well as meningeal inflammation score (**c**) of young (*n* = 15) and aged (*n* = 14) mice at the time of death caused by intracerebral infection with *S. pneumoniae* serotype 3. Symbols represent values of individual mice, and bars indicate medians, 25th and 75th percentiles. Meningeal inflammation score was calculated counting neutrophilic granulocytes in one high-power field (× 40 objective) on stained sections of three superficial meningeal regions and the hippocampal fissure and classified as follows: no leukocytes [score 0], < 10 leukocytes [score 1], 10–50 leukocytes [score 2], > 50 leukocytes [score 3]

The overall survival after intracerebral infection with *S. pneumoniae* serotype 3 did not differ between young (mortality 38%) and aged (mortality 35%) mice (Fig. 1: log-rank test, *P* = 0.98). Both groups lost weight during the disease progress. Immediately before infection, weight in young and aged mice was 24 ± 3 g and 33 ± 5.8 g (mean ± standard deviation), respectively. There was no significant difference in weight loss between young (0.9 ± 0.6 g) and aged (1.2 ± 0.8 g) mice (difference weight 0 to 24 h after infection, mean ± standard deviation, Student’s *t*-test, *P* = 0.077). The time elapsing before symptoms of disease occurred (clinical score 1) did not differ between young and aged mice (median [25./75. percentile]: young 36 [12/48] h versus aged 36 [18/36] h, Mann-Whitney *U*-test, *P* = 0.79). At 30 h after infection, 30.6% of the aged and 22% of the young mice showed bacterial blood concentrations above the detection limit of 1000 CFU/ml blood as a sign of septicemia, with aged mice reaching up to a maximum of 10^8^ CFU/ml blood compared to a maximum of 9 × 10^4^ CFU/ml blood of the young mice. Mann-Whitney *U*-test showed no significant differences in the concentrations of *S. pneumoniae* serotype 3 in blood 30 h after infection between young and aged mice (median [25./75. percentile]: young < 1000 [< 1000/< 1000] CFU/ml blood versus aged < 1000 [< 1000/8750] CFU/ml blood, *P* = 0.19). By the time of death due to infection, no differences between young and aged mice concerning the bacterial concentrations of *S. pneumoniae* serotype 3 in spleen (Fig. 2a, median [25./75. percentile]: young 3 × 10^5^ [1 × 10^5^/7 × 10^5^] versus aged 3.5 × 10^5^ [6.5 × 10^4^/8.5 × 10^5^] CFU/ml, Mann-Whitney *U*-test, *P* = 0.90) and in cerebellum (Fig. 2b, median [25./75. percentile]: young 1 × 10^8^ [6 × 10^7^/7 × 10^8^] versus aged 7.5 × 10^7^ [2 × 10^7^/2.5 × 10^8^] CFU/ml, Mann-Whitney *U*-test, *P* = 0.10) were detected. 15 days after infection, in all surviving young (*n* = 26) and surviving aged (n = 26) mice bacterial concentrations in spleen and cerebellum were below the detection limit of 100 CFU/ml. Comparing the invasion of neutrophilic granulocytes into the subarachnoid space using the meningeal inflammation score by the time of death of acute infection, there was no difference between young and aged mice (Fig. 2c, median [25./75. percentile]: young 1.6 [1.4/2] versus aged 1.6 [1.2/2], Mann-Whitney *U*-test, *P* = 0.61). 15 days after infection, 3 of the young and 6 of the aged surviving mice still showed low numbers of leukocytes in the subarachnoid space (maximum meningeal inflammation score: 1 in young and 0.4 in aged mice). However, there was no significant difference in the meningeal inflammation score between surviving young and surviving aged mice 15 days after infection (median [25./75. percentile]: young 0 [0/0] versus aged 0 [0/0], Mann-Whitney *U*-test, *P* = 0.34). In general, there was a significant correlation between the meningeal inflammation score and the bacterial concentration in the cerebellum in young (*r* = 0.9, *P* < 0.0001) as well as in aged (*r* = 0.87, *P* < 0.0001) mice.

## Discussion

The bacterium *S. pneumoniae* is the most common cause of bacterial meningitis in adults. The incidence of pneumococcal meningitis is four times higher in persons ≥60 years compared to persons aged 2 to 29 years, and mortality is approximately twice as high in patients ≥60 years compared to younger patients (37% vs. 18%) [4, 6]. In a Danish retrospective study of 464 adult patients with invasive pneumococcal disease, serotype 3 accounted for 24 cases (5.3%). 12 of these 24 patients, i.e. 50%, died. After adjustment for other parameters of disease severity, bacteremic pneumonia, bacteremia with unknown focus, meningitis and otitis media caused by *S. pneumoniae* serotype 3 was associated with an increased mortality (relative risk 2.54 compared to other *S. pneumoniae* serotypes) [9].

In order to understand the more severe course of the disease of pneumococcal meningitis in elderly patients, we aimed at establishing a murine model which mimics the clinical situation and thus helps to understand the underlying causes. We previously succeeded to do so with another important pathogen of bacterial meningitis in the elderly, *E. coli*. In our geriatric mouse model of *E. coli* meningitis, aged mice showed a higher mortality, an impaired elimination of bacteria, a faster systemic spread of bacteria and an impaired systemic inflammatory response. In these previous experiments, the numbers of microglia and of infiltrating neutrophilic granulocytes in the subarachnoid space after intracerebral *E. coli* K1 infection did not differ between young and aged mice, but there was a significant impairment of phagocytosis of *E. coli* K1 and a decreased release of inflammatory cytokines by aged compared to young microglia and macrophages in vitro [7]. In contrast to these previous results, we did not find any differences in the course of bacterial meningitis caused by *S. pneumoniae* serotype 3 between young and aged mice. Although in our mouse models the intracerebrally injected dose of *E. coli* K1 (10^5^ CFU) was much higher than that of *S. pneumoniae* serotype 3 (50–70 CFU), mortality, time until death as well as meningeal inflammation scores, and bacterial concentrations in cerebellum and spleen were similar in young mice in both models [7]. These results underline the higher virulence of *S. pneumoniae* serotype 3 compared to *E. coli* K1.

The most important determinant of the virulence of *S. pneumoniae* is its polysaccharide capsule [10]. Possession of a capsule is essential for causing meningitis [11]. Serotype 6B *S. pneumoniae* strains, which maintained a thick capsule in the cerebrospinal fluid, caused a more severe disease than serotype 7F in the same genetic background, which had a much thinner capsule [11]. *S. pneumoniae* serotype 3 has a thicker capsule compared to most other *S. pneumoniae* strains [12, 13] which protects against phagocytosis and might account for its high virulence [14]. In neutrophils, increase in capsule size of *S. pneumoniae* is associated with a decreased rate of phagocytosis [15]. The *S. pneumoniae* serotype 3 strain HB565 was resistant to phagocytosis by cells of the microglial BV2-cell line [16]. The *S. pneumoniae* serotype 3 used in the present experiments (strain A; [17]) produced very large, mucoid colonies on blood-agar plates, and capsular expression was stable during infection in experimental animals. *S. pneumoniae* serotype 2 D39, which has a thinner capsule compared to *S. pneumoniae* serotype 3 [13] was phagocytosed by primary murine microglial cells of young mice only in small numbers [18], whereas phagocytosis of the unencapsulated *S. pneumoniae* strain R6 and also *E. coli* K1 was considerably higher [18, 19]. Microglia as well as perivascular and meningeal macrophages, the immune cells representing the first line of defense of the CNS, undergo age-related changes concerning their phagocytic ability [7, 20]. Aged microglial cells and macrophages show an impaired ability to phagocytose *E. coli* K1 going along with an impaired elimination of bacteria and an increased mortality in aged mice after intracerebral infection with *E. coli* K1 [7]. This age-related decline of phagocytosis of pathogens seems to have less impact on the outcome of an infection caused by a hardly phagocytable bacterium with a thick polysaccharide capsule such as *S. pneumoniae* serotype 3. In accordance with our findings, Esposito et al. showed, that a deposition of viable *S. pneumoniae* serotype 3 in the lower respiratory tract resulted in equal mortality of young and aged C57BL/6 mice [21]. These observations suggest that in the presence of a thick capsule with a high content of capsular polysaccharide neither young nor old phagocytes can control the infection, once pneumococci have reached physiologically sterile body sites, and correspond to the high mortality of invasive *S. pneumoniae* infections caused by serotype 3 strains [9].

Conversely there may be a difference between the resistance to *S. pneumoniae* infection of old and young animals, when the pneumococcal capsule is thinner and contains smaller amounts of polysaccharide. Intratracheal infection with *S. pneumoniae* serotype 4 showed that aged Balb/cBy mice had a higher mortality and higher bacterial concentrations compared to young Balb/cBy mice [22]. Furthermore, pneumococcal colonization was prolonged and clearance of pneumococci was delayed in the higher respiratory tract in aged compared to young C57BL/6 mice after nasal instillation of *S. pneumoniae* serotype 6B [23].

In summary, when a highly virulent *S. pneumoniae* type 3 strain was used, no difference was found in the course of meningitis in old and young mice after intracerebral inoculation. Data from other models of infection generated with less virulent serotypes of *S. pneumoniae* suggest that these less aggressive serotypes may be more suitable to study differences in the age-related susceptibility towards *S. pneumoniae* infections.

## Conclusions

In contrast to results from our geriatric mouse model of *E. coli* meningitis, where we found a higher mortality and an impaired elimination of bacteria in aged mice compared to young mice, we did not find any significant differences between aged and young mice concerning the disease course after intracerebral infection with the pathogen *S. pneumoniae* serotype 3. We conclude that the age-related decline of the phagocytic capacity of microglia and macrophages has only a minor impact on the course of CNS infections caused by pathogens with thick capsules such as *S. pneumoniae* serotype 3. A larger panel of *S. pneumoniae* serotypes should be studied in vivo and in vitro in order to better elucidate age-related susceptibility to this life-threatening pathogen.

## Abbreviations

CAE: Chloracetate esterase
CFU: Colony forming units
CNS: Central nervous system
*E. coli*: *Escherichia coli*
*S. pneumoniae*: *Streptococcus pneumoniae*
